# PAR4 (Protease-Activated Receptor 4) Antagonism With BMS-986120 Inhibits Human Ex Vivo Thrombus Formation

**DOI:** 10.1161/ATVBAHA.117.310104

**Published:** 2018-01-24

**Authors:** Simon J. Wilson, Fraz A. Ismat, Zhaoqing Wang, Michael Cerra, Hafid Narayan, Jennifer Raftis, Timothy J. Gray, Shea Connell, Samira Garonzik, Xuewen Ma, Jing Yang, David E. Newby

**Affiliations:** From the British Heart Foundation Centre for Cardiovascular Science (S.J.W., D.E.N.), Medical Research Council Centre for Inflammation Research (J.R., S.C.), and Edinburgh College of Medicine (T.J.G.), University of Edinburgh, United Kingdom; Bristol Myers Squibb, Princeton, NJ (F.A.I., Z.W., M.C., S.G., X.M., J.Y.); and Royal Infirmary of Edinburgh, United Kingdom (H.N.).

**Keywords:** antiplatelet, human, novel, protease-activated receptor 4, thrombosis

## Abstract

**Objective—:**

BMS-986120 is a novel first-in-class oral PAR4 (protease-activated receptor 4) antagonist with potent and selective antiplatelet effects. We sought to determine for the first time, the effect of BMS-986120 on human ex vivo thrombus formation.

**Approach and Results—:**

Forty healthy volunteers completed a phase 1 parallel-group PROBE trial (Prospective Randomized Open-Label Blinded End Point). Ex vivo platelet activation, platelet aggregation, and thrombus formation were measured at 0, 2, and 24 hours after (1) oral BMS-986120 (60 mg) or (2) oral aspirin (600 mg) followed at 18 hours with oral aspirin (600 mg) and oral clopidogrel (600 mg). BMS-986120 demonstrated highly selective and reversible inhibition of PAR4 agonist peptide (100 μM)-stimulated P-selectin expression, platelet-monocyte aggregates, and platelet aggregation (*P*<0.001 for all). Compared with pretreatment, total thrombus area (μm^2^/mm) at high shear was reduced by 29.2% (95% confidence interval, 18.3%–38.7%; *P*<0.001) at 2 hours and by 21.4% (9.3%–32.0%; *P*=0.002) at 24 hours. Reductions in thrombus formation were driven by a decrease in platelet-rich thrombus deposition: 34.8% (19.3%–47.3%; *P*<0.001) at 2 hours and 23.3% (5.1%–38.0%; *P*=0.016) at 24 hours. In contrast to aspirin alone, or in combination with clopidogrel, BMS-986120 had no effect on thrombus formation at low shear (*P*=nonsignificant). BMS-986120 administration was not associated with an increase in coagulation times or serious adverse events.

**Conclusions—:**

BMS-986120 is a highly selective and reversible oral PAR4 antagonist that substantially reduces platelet-rich thrombus formation under conditions of high shear stress. Our results suggest PAR4 antagonism has major potential as a therapeutic antiplatelet strategy.

**Clinical Trial Registration—:**

URL: http://www.clinicaltrials.gov. Unique identifier: NCT02439190.

## Abstract 2

Supplemental Digital Content is available in the text.

Platelets are central to thrombus formation, the leading cause of global mortality.^1^ Antiplatelet drugs are of proven benefit for the treatment and prevention of atherothrombotic events in many clinical settings, but despite the introduction of newer agents in the last decade, important limitations persist. Aspirin and P2Y12 antagonists, the current standard of care oral antiplatelet agents in patients with acute coronary syndrome, stroke, and peripheral arterial disease, prevent thromboxane A2 and ADP platelet activation, respectively.^2–7^ However, neither is effective against thrombin, the most potent of all platelet agonists,^8^ and both are associated with an increased incidence of bleeding that restricts their use in sensitive populations (eg, elderly, cerebrovascular disease) and reduces their net clinical benefit.^5,9–12^ Thus, despite contemporary antiplatelet pharmacotherapy, many patients remain at high risk of future atherothrombotic events,^5–7,10,13,14^ and there is a clear need for newer agents that can provide equivalent (or superior) antithrombotic efficacy with an improved safety profile.

**See accompanying editorial on page 287**

In recent years, PAR4 (protease-activated receptor 4) antagonism has emerged as promising new antiplatelet strategy. PAR4 is a G-protein coupled receptor expressed on the platelet surface that together with PAR1 (protease-activated receptor 1) is responsible for thrombin-mediated platelet activation and aggregation.^15^ Thrombin has a key role in the coagulation cascade, but by targeting the platelet receptor rather than the protease, this avoids directly interfering with thrombin-induced fibrin production. PAR1 has greater affinity for thrombin than PAR4, but despite early clinical promise, the addition of vorapaxar (the only licensed PAR1 antagonist) to standard care failed to meet its primary efficacy outcome in patients with acute coronary syndrome and was associated with an excess of major bleeding, especially intracranial hemorrhage, in phase3 clinical trials.^13,16^ PAR4 was originally thought to simply provide some redundancy to PAR1 platelet signaling at high thrombin concentrations.^17^ However, due to differences in activation kinetics and downstream pathways, it is now evident that PAR1 and PAR4 have distinct and complementary roles in the early and late phases of platelet activation and aggregation, respectively.^18–20^ PAR1 activation is brisk but transient and requires input from the P2Y12-PI3K (phosphatidylinositol 3-kinase) pathway to maintain platelet aggregation.^19,20^ In contrast, PAR4 is activated at higher thrombin concentrations and induces a slow but prolonged intracellular signal that acts independently to sustain irreversible aggregation.^17,18,20^ Furthermore, PAR4 activation occurs after ADP secretion, and thrombin depends on PAR4 but not PAR1 to induce full platelet spreading.^21^ Thus, several lines of evidence indicate that while PAR1 and other agonist-signaling pathways may have important roles in initiating platelet activation, the primary function of PAR4 appears to be in sustaining irreversible platelet aggregation and thrombus propagation. This suggests that selectively targeting PAR4-mediated thrombin activity may protect against occlusive thrombus formation while avoiding interfering with hemostatic platelet responses to the same extent as PAR1 antagonists and other antiplatelet agents.^22^

BMS-986120 is a first-in-class, oral, highly selective, and reversible PAR4 antagonist antiplatelet agent. In preclinical animal models, BMS-986120 demonstrated potent antithrombotic activity with a substantially wider therapeutic window when compared with clopidogrel.^22^ The purpose of the present phase 1 parallel-group PROBE trial (Prospective Randomized Open-Label Blinded End Point) was to build on these observations and examine for the first time, the antiplatelet and antithrombotic effects of BMS-986120 in humans using a translational model of ex vivo thrombosis. We determined whether reductions in thrombus formation were driven by a decrease in platelet-rich or fibrin-rich thrombus formation and whether these effects were greater under rheological conditions of low or high shear stress.

## Materials and Methods

Materials and Methods are available in the online-only Data Supplement.

## Results

All 40 volunteers (81 volunteers were screened) completed the study in full. The demographics and baseline characteristics of study volunteers were similar in the 2 treatment groups (Table). BMS-986120 was well tolerated with no clinically significant effect on any of the biochemical, hematologic, coagulation, physical, or ECG safety assessments conducted throughout the study (Table I in the online-only Data Supplement). There were no serious adverse events. One episode of minor bleeding was reported. This occurred 12 hours after aspirin administration, self-resolved, and did not recur.

**Table.**
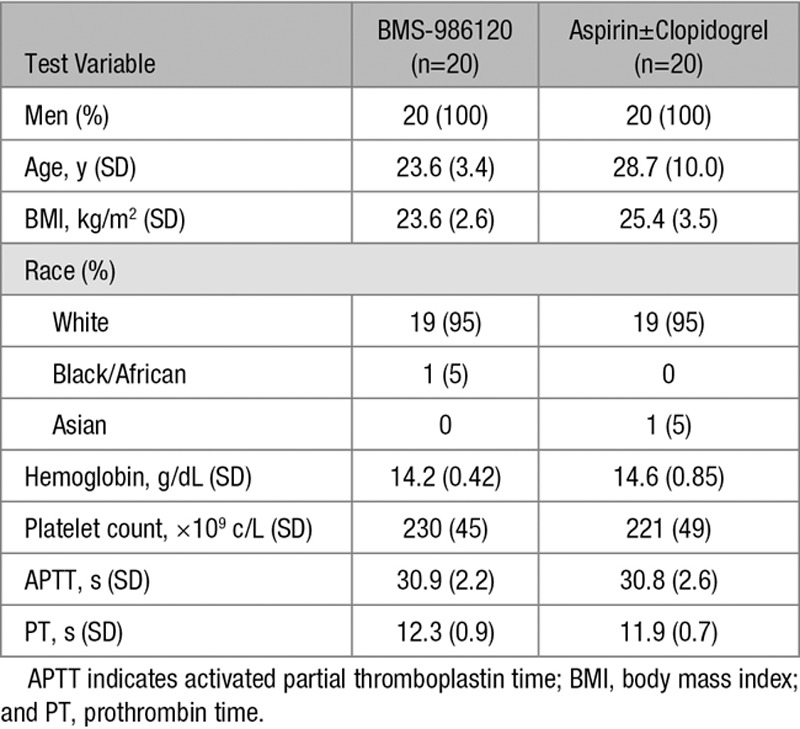
Baseline Characteristics of Study Volunteers

### Pharmacokinetic Profile of Oral BMS-986120

**Figure 1.**
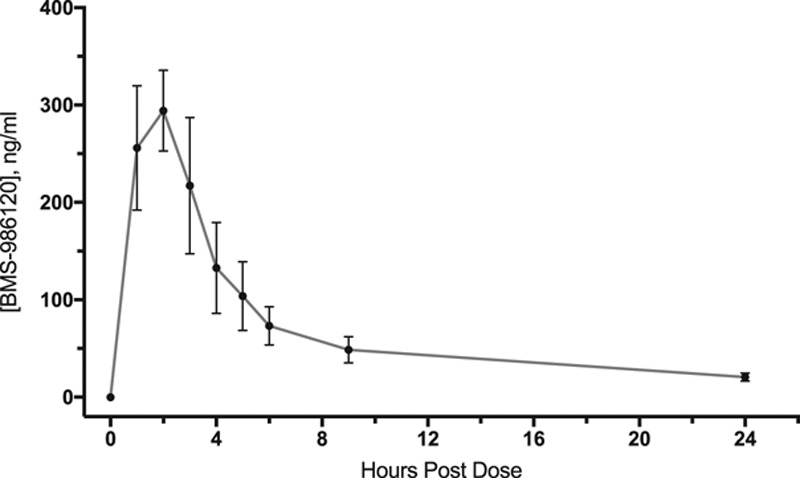
Pharmacokinetics of BMS-986120. BMS-986120 was rapidly absorbed with a half-life of 4 h. Data shown are mean plasma concentrations of BMS-986120 (±95% confidence intervals) after administration of a single oral 60-mg dose.

BMS-986120 was rapidly absorbed with peak plasma concentrations occurring at 2 hours (255±136 ng/mL; Figure 1). Plasma concentrations of BMS-986120 were halved by 4 hours (133±100 ng/mL) and <10% of the peak concentration by 24 hours (21±9 ng/mL).

### Effect of BMS-986120 on Platelet Activation and Aggregation

**Figure 2.**
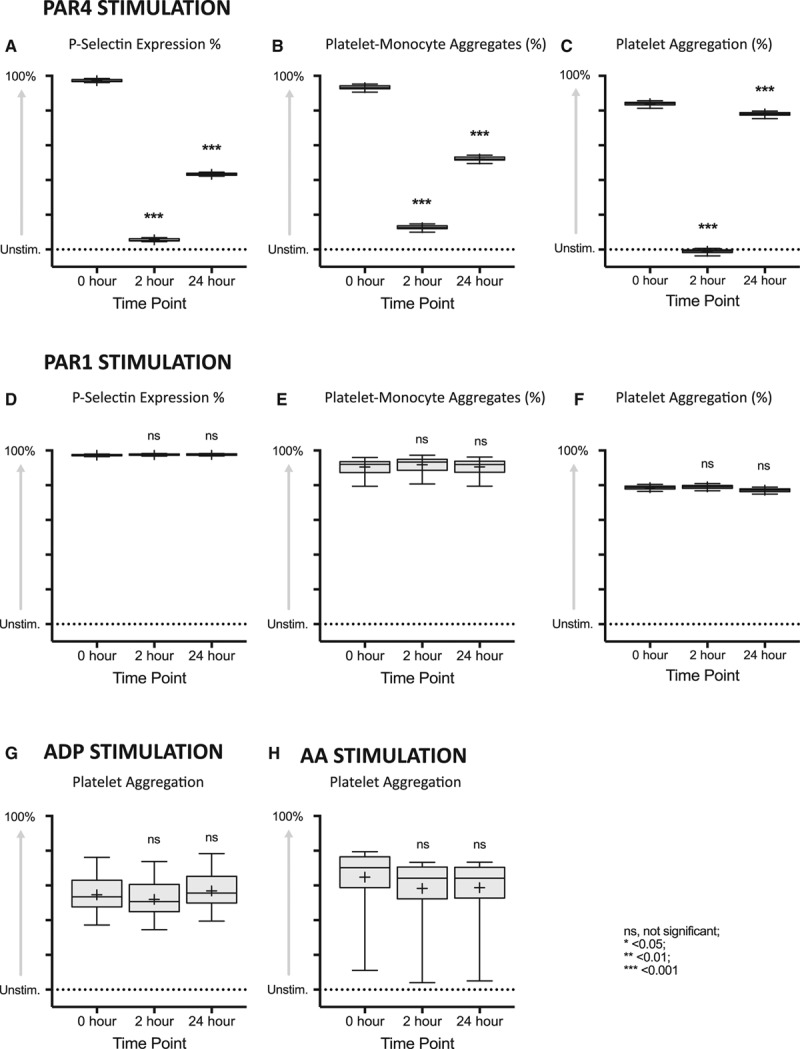
BMS-986120 demonstrated highly selective, potent, and reversible inhibition of PAR (protease-activated receptor) 4-stimulated platelet activation and aggregation. Box plots of platelet activation and aggregation in response to (**A–C**) PAR4 Agonist peptide (AP; 100 μM), (**D** and **E**) PAR1 AP (100 μM), (**F**) PAR1 AP (25 μM), (**G**) ADP (10 μM), and (**H**) arachidonic acid (AA; 5 mmol/L), in volunteers randomized to BMS-986120. Data shown are the adjusted mean (+) normalized to unstimulated values. The line within the box represents the median, upper and lower edges of the box represent the 75th and 25th percentiles, and upper and lower whiskers represent the 95th and 5th percentiles. Statistical comparisons (least significance difference test) vs 0 h are represented above each plot. ns indicates nonsignificant. *P<0.05, **P<0.01, ***P<0.001.

BMS-986120 demonstrated strong and reversible inhibition of PAR4 agonist peptide (AP; 100 μM)-stimulated platelet activation and aggregation (*P*<0.001 for all). Compared with pretreatment, PAR4 AP-stimulated increases in platelet P-selectin expression (%), platelet-monocyte aggregates (%), and platelet aggregation (%) were reduced by 91.7% (95% confidence interval [CI], 81.0–102.4), 80.6% (95% CI, 68.6%–92.6%), and 85.0% (95% CI, 82.0–88.1) at 2 hours and by 53.9% (95% CI, 43.2%–64.7%), 41.1% (95% CI, 28.9%–53.2%), and 6.0% (95% CI, 2.9%–9.0%) at 24 hours (*P*<0.001 for all; Figure 2). Plasma concentrations of BMS-986120 correlated with P-selectin expression (ρ=−0.87), platelet-monocyte aggregates (ρ=−0.88), and platelet aggregation (ρ=−0.82; *P*<0.001 for all; Figure III in the online-only Data Supplement). There was no effect on PAR1 AP, ADP, or arachidonic acid platelet responses (*P*=nonsignificant [ns] for all; Figure 2).

### Effect of Aspirin±Clopidogrel on Platelet Aggregation

Aspirin administration reduced arachidonic acid-stimulated platelet aggregation by 74.5% (95% CI, 71.6%–77.3%; *P*<0.001). In combination with clopidogrel, aspirin reduced arachidonic acid-stimulated platelet aggregation by 73.7% (95% CI, 70.9%–76.5%; *P*<0.001) and ADP-stimulated platelet aggregation by 41.9% (95% CI, 35.2%–48.7%; *P*<0.001), respectively (Figure IV in the online-only Data Supplement).

### Effect of BMS-986120 on Ex Vivo Thrombus Formation

**Figure 3.**
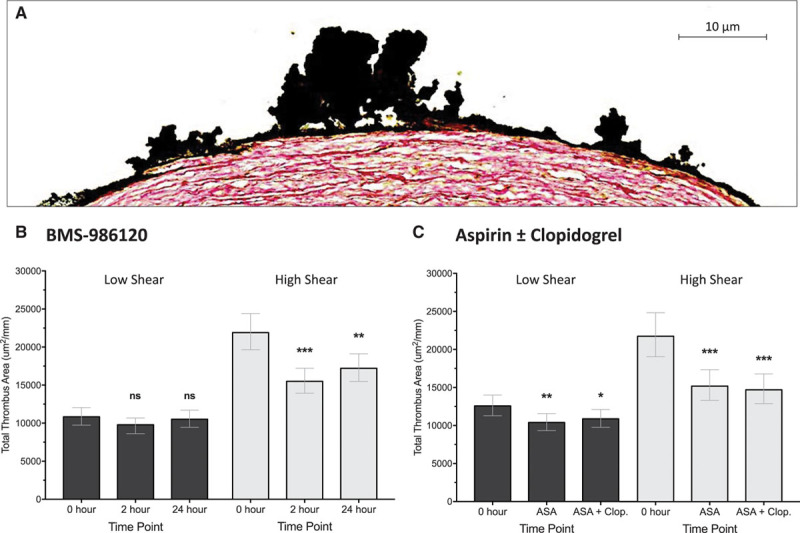
BMS-986120 reduced thrombus formation at high shear but not at low shear. **A**, Representative image of porcine aortic media exposed to human blood at high shear stained to quantify total thrombus area. Sections were stained with polyclonal goat antihuman fibrin(ogen) antibody and CD61 monoclonal mouse antihuman antibody before treatment with 3,3′-diaminobenzidine substrate chromogen. Sections were then counterstained with a modified Masson trichrome (hematoxylin and sirius red, 0.1%). Effect of (**B**) BMS-986120 and (**C**) aspirin (ASA)±clopidogrel (Clop.) on total thrombus area at high and low shear. Statistical comparisons (least significance difference test) vs 0 h are represented above each plot. ns indicates nonsignificant. *P<0.05, **P<0.01, ***P<0.001.

BMS-986120 reduced total thrombus formation at high shear (*P*<0.001) but not at low shear (*P*=ns; Figure 3). Compared with pretreatment, total thrombus area (μm^2^/mm) at high shear was reduced by 29.2% (95% CI, 18.3%–38.7%; *P*<0.001) at 2 hours and by 21.4% (95% CI, 9.3%–32.0%; *P*=0.002) at 24 hours. Plasma concentrations of BMS-986120 correlated with total thrombus formation at high shear (ρ=−0.47; *P*<0.001) but not at low shear (ρ=−0.18; *P*=ns; Figure III in the online-only Data Supplement).

**Figure 4.**
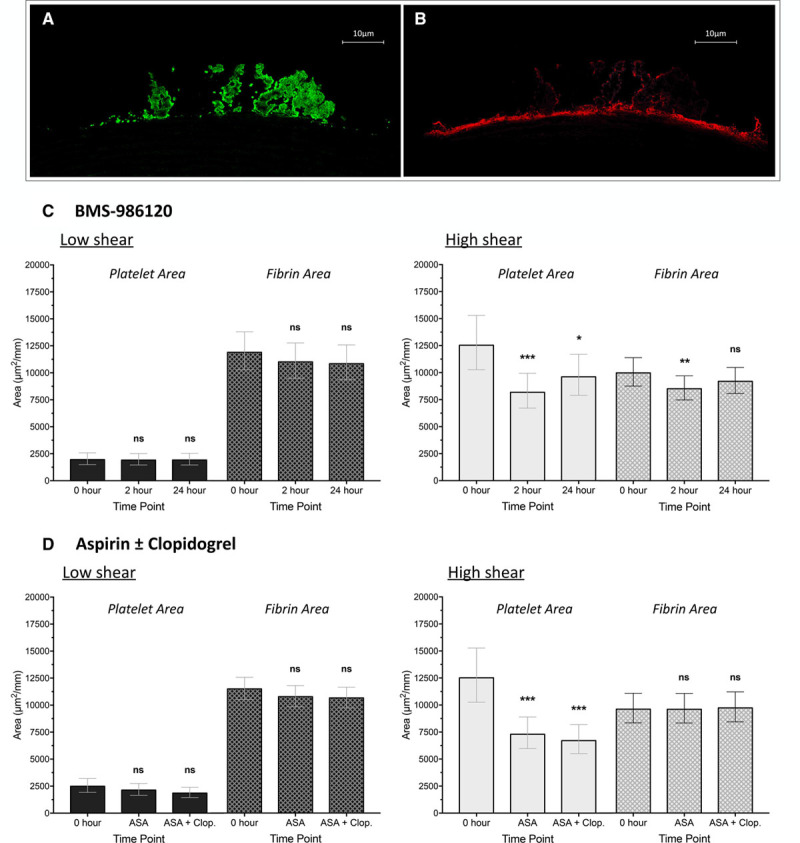
Reductions in thrombus formation were driven by a decrease in platelet-rich thrombus formation. Representative image of thrombus formed at high shear stained to allow quantification of (**A**) platelet-rich and (**B**) fibrin-rich thrombus area. Sections were stained with polyclonal goat antihuman fibrin(ogen) antibody and CD61 monoclonal mouse antihuman antibody before counterstaining with tyramide Cy3 (cyanine 3) and FITC (fluorescein isothiocyanate). Effect of (**C**) BMS-986120 and (**D**) aspirin (ASA)±clopidogrel (Clop.) on platelet and fibrin deposition at low and high shear. Data shown are adjusted means±95% confidence intervals. Statistical comparisons (least significance difference test) vs 0 h are represented above each plot. ns indicates nonsignificant. *P<0.05, **P<0.01, ***P<0.001.

Reductions in total thrombus area were driven by a decrease in platelet deposition (Figure 4). At high shear, platelet-rich thrombus area was reduced by 34.8% (95% CI, 19.3%–47.3%; *P*<0.001) at 2 hours and 23.3% (95% CI, 5.1%–38.0%; *P*=0.016) at 24 hours. Reductions in fibrin-rich thrombus area at 2 (−14.7%; 95% CI, −22.5% to −6.2%; *P*=0.002) and 24 hours (−7.9%; 95% CI, −16.3% to 1.4%; *P*=0.09) were small by comparison. BMS-986120 had no effect on either platelet-rich or fibrin-rich thrombus formation at low shear (*P*=ns for all).

### Effect of Aspirin±Clopidogrel on Ex Vivo Thrombus Formation

Aspirin and aspirin in combination with clopidogrel both reduced thrombus formation at high and low shear, also driven by decrease in platelet-rich thrombus. Aspirin reduced total thrombus area and platelet-rich thrombus area by 30.2% (95% CI, 15.6%–42.2%; *P*<0.001) and 41.7% (95% CI, 22.9%–56.0%; *P*<0.001), respectively, and by 32.4% (95% CI, 18.3%–44.0%; *P*<0.001) and 46.4% (95% CI, 29.1%–59.5%; *P*<0.001), respectively, when used in combination with clopidogrel.

In contrast to BMS-986120, aspirin and aspirin in combination with clopidogrel both reduced total thrombus area at low shear (−17.4%; 95% CI, −27.0% to −6.5%; *P*=0.003 and −13.5%; 95% CI, −23.6% to −2.1%; *P*=0.02). There was no effect on fibrin-rich thrombus deposition at low or high shear (*P*=ns for all).

## Discussion

In this phase 1 PROBE designed clinical trial, we have shown for the first time that PAR4 antagonism with BMS-986120 reduces ex vivo human thrombus formation under conditions representative of deep arterial injury in a stenosed coronary artery. BMS-986120 demonstrated selective and reversible antiplatelet effects with concentration-dependent inhibition of thrombus formation and PAR4 AP-stimulated platelet activation and aggregation. Our results provide further insights into the role of PAR4 in human thrombogenesis and raise major promise for BMS-986120 as an antiplatelet agent in the treatment and prevention of arterial thrombosis.

Assessment of the antiplatelet and antithrombotic effects of PAR4 inhibition has previously been limited by a lack of compound specificity and availability.^23–25^ In comparison with earlier compounds, including P4pal-10, YD-3, and its derivative ML354,^25–27^ BMS-986120 has antiplatelet activity against α thrombin, demonstrated greater potency and selectivity of effect in preclinical and phase 1 studies of platelet inhibition, and is the first orally bioavailable PAR4 antagonist.^22,28^ In the present study, a single dose of BMS-986120 resulted in near complete inhibition of PAR4 AP-stimulated platelet activation and aggregation at 2 hours, with a return toward baseline at 24 hours. Importantly, there was no effect on PAR1 AP, ADP, or arachidonic acid-stimulated platelet activity. Our data, therefore, add to previous studies indicating that BMS-986120 is a highly selective and reversible antiplatelet agent with potent activity against PAR4-stimulated platelet activation and aggregation in humans.

The antithrombotic effects of BMS-986120 in humans were examined using the Badimon perfusion chamber—a well validated model for measuring ex vivo thrombus formation in humans.^29–36^ Using the same model and under the same flow conditions, previous studies in healthy volunteers have demonstrated reductions in high shear thrombus formation of 18.7% after a single 300-mg oral dose of clopidogrel, 28% with a 60-mg oral dose of edoxaban and 56% with extracorporeal coadministration of tirofiban (50 ng/mL).^29,36,37^ In the present study, a single dose of BMS-986120 (60 mg) reduced high shear thrombus formation by nearly a third. This is consistent with preclinical animal data^22,23^ and comparable with reductions in thrombus formation we observed with high loading doses of aspirin and clopidogrel. Importantly, therefore, we have shown that oral PAR4 antagonism with BMS-986120 substantially reduces ex vivo human thrombus formation. Moreover, reductions were similar in magnitude to clinically approved antiplatelet agents suggesting a high probability of in vivo antithrombotic efficacy.

BMS-986120 seemed to have less of an effect on thrombus formation at low shear than either aspirin alone or aspirin in combination with clopidogrel. Although further studies are required to confirm whether PAR4 antagonism is more selective for inhibiting thrombus formation at high shear than existing agents, distinct mechanisms of platelet aggregation are known to operate under different rheological conditions.^38,39^ Low shear rates reflect flow conditions found in patent epicardial arteries and some veins, whereas the majority of atherothrombotic events invariably occur at areas of high shear stress seen in diseased arteries.^40,41^ Indeed, most myocardial infarctions arise from stenotic atherosclerotic plaques with rheological conditions comparable with those in our high shear chamber.^42–44^ Antiplatelet agents that are more selective for inhibiting thrombus formation at high shear may allow at-risk vascular beds to be targeted with greater specificity. Given many treatment-related bleeding events are likely to occur from vessels with low shear rates,^45–49^ this could facilitate a wider safety profile.

As expected from an antiplatelet agent, reductions in thrombus were driven by a decrease in platelet deposition; however, there was also a small but significant reduction in fibrin-rich thrombus formation. PAR4 is reported to be the predominant platelet PAR responsible for phosphatidylserine exposure, microparticle shedding, and thrombin generation.^50^ Our results add to these studies, indicating that PAR4 may have a role in platelet procoagulant activity during ex vivo human thrombus formation. Whether this is beneficial or not is uncertain, but it is worth noting BMS-986120 was not associated with an increase in coagulation assay times, and no bleeding-related clinical findings or adverse events were reported in a previous phase 1 single- and multiple-ascending dose study.^28^

PAR4 is expressed within the vasculature, and PAR4 antagonism may, in addition to protecting against thrombosis, serve to limit vascular complications in at-risk patients. Human vascular smooth muscle cells upregulate PAR4 in response to glucose,^51^ and elevated expression of PAR4 has been reported in the tunica media of atherectomy and saphenous vein tissue from patients with diabetes mellitus.^51^ Moreover, PAR4 deficiency protected against excessive remodeling induced by carotid artery ligation in streptozotocin-diabetic mice.^52^ PAR4, therefore, seems to be a key regulator of exaggerated intimal thickening in diabetes mellitus, and future studies examining the antiproliferative potential of PAR4 antagonism would be of significant therapeutic interest.

Our study has some limitations. First, although the exposed porcine aortic media presents many of the common constituents of a disrupted atherosclerotic plaque, including type I collagen, it may not contain tissue factor (TF).^53–55^ TF activates the coagulation cascade and is an important contributor to thrombogenicity.^56,57^ Nevertheless, this does not overly limit our model for the assessment of thrombosis because binding of blood-borne circulating TF is sufficient to allow activation of the coagulation cascade and thrombus propagation.^53,54,58–60^ Indeed, previous studies have demonstrated that thrombus formed from human blood perfused over exposed porcine tunica media (devoid of TF) stains heavily for TF.^53,54^ Second, we assessed a single oral dose of BMS-986120 and did not explore the effect of prolonged BMS-986120 administration on thrombus formation, such as would occur with the secondary prevention of myocardial infarction and stroke. However, because this was the phase 1 trial designed to examine the antithrombotic effects of oral PAR4 antagonism in humans for the first time, we felt our study design was appropriate. Third, BMS-986120 was dosed in isolation, and future studies to determine the antiplatelet and antithrombotic effects of PAR4 antagonism in combination with current agents would be of interest. Finally, although no episodes of bleeding occurred in volunteers administered BMS-986120 and BMS-986120 was not associated with an increase in bleeding times in a previous phase 1 safety and tolerability study,^28^ the safety profile of PAR4 antagonism in humans remains to be defined.

In conclusion, we have demonstrated that PAR4 antagonism with BMS-986120—a highly selective and reversible oral PAR4 antagonist—substantially reduces ex vivo thrombus formation in healthy volunteers under conditions of high shear stress. BMS-986120 was well tolerated with no change in coagulation assays or serious adverse events. Given the potential hemostatic sparing effects of PAR4 antagonism, our results suggest that BMS-986120 has major potential as a novel antiplatelet agent and that further investigation in clinical trials is warranted.

## Acknowledgments

We are grateful to the Histology Department of the Queen’s Medical Research Institute (Edinburgh, United Kingdom) for their support and expertise in conducting this study. Edinburgh Clinical Research Facility is supported by the National Health Service Research Scotland through National Health Service Lothian Health Board.

## Sources of Funding

This study was funded by Bristol-Myers Squibb.

## Disclosures

Dr Newby is supported by the British Heart Foundation (significant: CH/09/002, RM/13/2/30158, and RE/13/3/30183) and is the recipient of a Wellcome Trust Senior Investigator Award (significant: WT103782AIA). Drs Wilson (moderate) and Newby (moderate) were supported by, and have undertaken consultancy for, Bristol-Myers Squibb. Z. Wang (significant), M. Cerra (significant), and Drs Ismat (significant), Garonzik (significant), Ma (significant), and Yang (significant) are employed by Bristol-Myers Squibb.

## Supplementary Material

## Notes

Highlights

- Inhibition of thrombin-mediated platelet activation through PAR4 (protease-activated receptor 4) antagonism represents a promising new antiplatelet strategy because of the potential for reduced bleeding.
- BMS-986120 is a first-in-class, oral, highly selective, and reversible PAR4 antagonist antiplatelet agent.
- A single dose of BMS-986120 substantially reduced ex vivo thrombus formation in healthy volunteers under conditions of high shear stress, driven by a reduction in platelet-rich thrombus deposition.
- Our results suggest PAR4 antagonism with BMS-986120 holds major promise as a novel antiplatelet strategy because of the potential for a wider therapeutic window in terms of antithrombotic efficacy and bleeding risk.
