# Supplementary material for: PAR4 (Protease-Activated Receptor 4) Antagonism With BMS-986120 Inhibits Human Ex Vivo Thrombus Formation
Source: Arterioscler Thromb Vasc Biol. 2018 Jan 24;38(2):448–56. doi: 10.1161/ATVBAHA.117.310104 (PMC5779320; doi:10.1161/ATVBAHA.117.310104)
Supplement: Supplementary file 1 [file atv-38-448-s001.pdf]

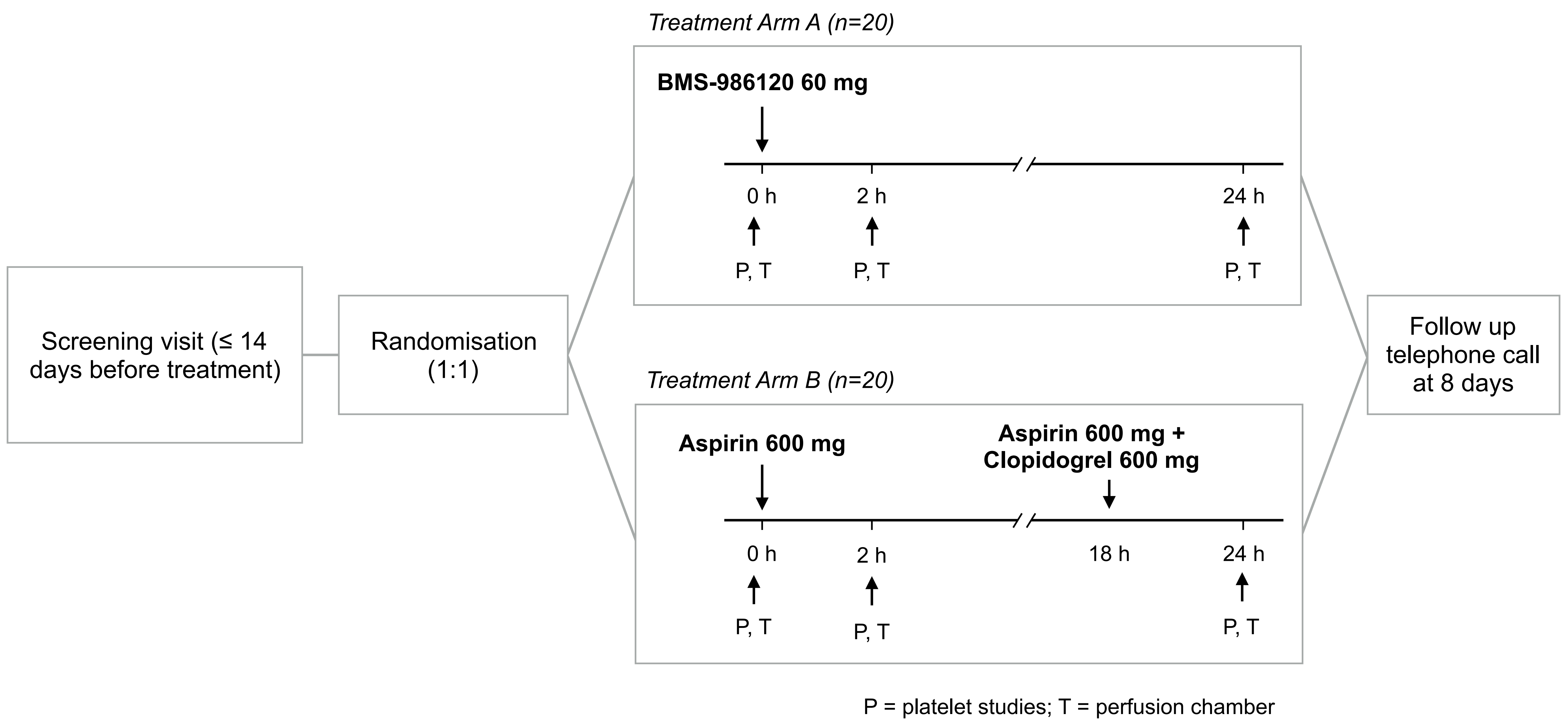

Figure I. Schematic overview of study design.

Table I. Safety Assessments

Test variable	BMS-986120 (n=20)			Aspirin ± Clopidogrel (n=20)		
	0 hour	*2 hour*	*24 hour*	*0 hour*	*ASA*	*ASA + Clop*
ALT, U/L (SD)	19.0 (6.1)	18.7 (6.2)	17.8 (6.4)**	26.3 (14.8)	25.9 (14.2)	25.4 (13.3)
AST, U/L (SD)	20.7 (4.6)	20.9 (5.7)	18.0 (3.6)***	23.6 (7.4)	23.4 (6.8)	21.0 (4.5)**
Bilirubin, mg/dL (SD)	0.70 (0.3)	0.80 (0.3)*	0.62 (0.3)	0.76 (0.2)	0.73 (0.2)	0.66 (0.2)**
Creatine Kinase, U/L (SD)	197 (190)	187 (188)	115 (83)***	186 (144)	171 (128)	108 (61)***
Urea, mmol/L (SD)	5.1 (0.96)	4.6 (0.76)**	4.1 (0.77)***	5.0 (1.0)	4.7 (0.93)	4.5 (0.98)**
Creatinine, mg/dL (SD)	0.83 (0.06)	0.81 (0.09)	0.82 (0.08)	0.89 (0.11)	0.86 (0.09)	0.86 (0.10)
Haemoglobin, g/dL (SD)	14.2 (0.4)	14.2 (0.4)	14.2 (0.3)	14.6 (0.8)	14.4 (0.8)	14.3 (1.3)
Platelet count, x10^9 c/L (SD)	229 (45)	227 (46)	228 (46)	221 (49)	220 (50)	216 (53)
APTT, seconds (SD)	30.9 (2.1)	30.3 (2.7)	30.4 (2.1)	30.8 (2.5)	30.3 (3.3)	30.1 (2.9)
PT, seconds (SD)	12.3 (0.9)	12.3 (0.9)	12.1 (0.8)	11.9 (0.7)	12.3 (0.7)**	12.1 (0.7)
QTCf interval, milliseconds (SD)	405 (11.8)	411 (17.9)	405 (15.9)	401 (16.3)	402 (13.4)	399 (10.8)

Data shown are the adjusted means with standard deviation. All significant differences (Least Significance Difference test) versus 0 hour are presented: * p<0.05, ** p<0.01, *** p<0.001. Abbreviations used: ASA, aspirin; Clo, clopidogrel. ALT, alanine transaminase; ASA, Aspirin; AST, aspartate transaminase; APTT, activated partial thromboplastin time; Clop, Clopidogrel; PT, prothrombin time; QTCf, QTC interval corrected for heart rate by Fridericia's formula; and SD, standard deviation

PAR4 AP Stimulated Platelet Responses

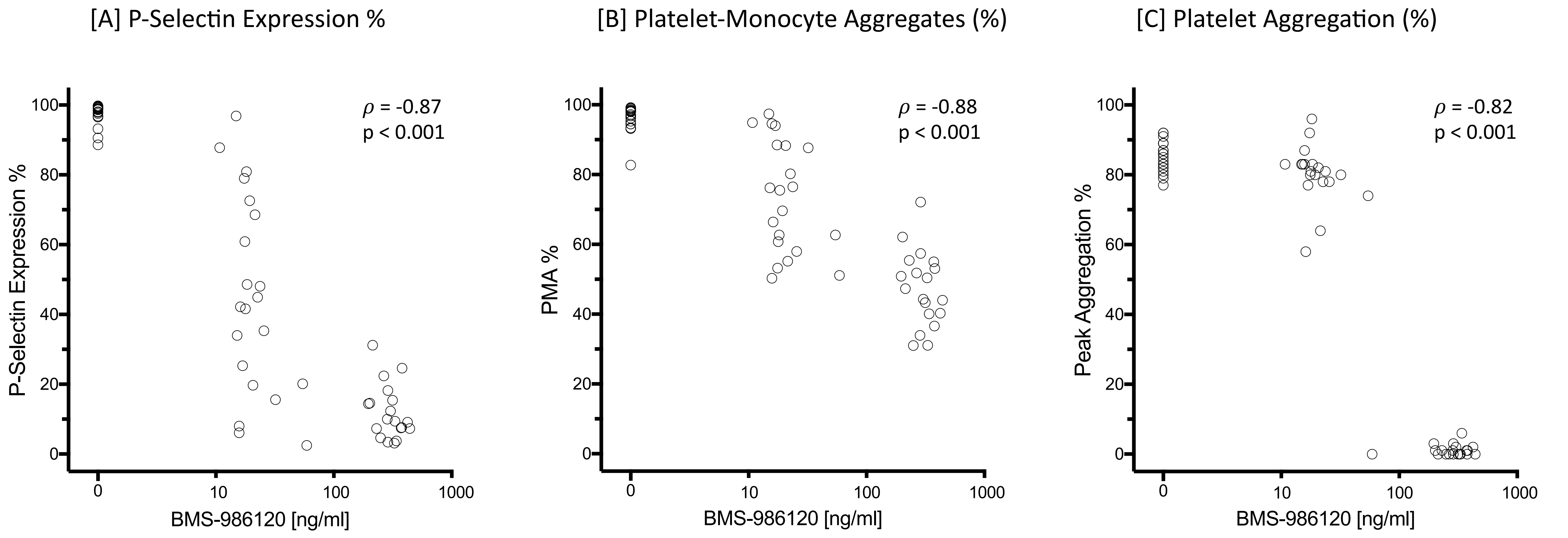

Total Thrombus Area

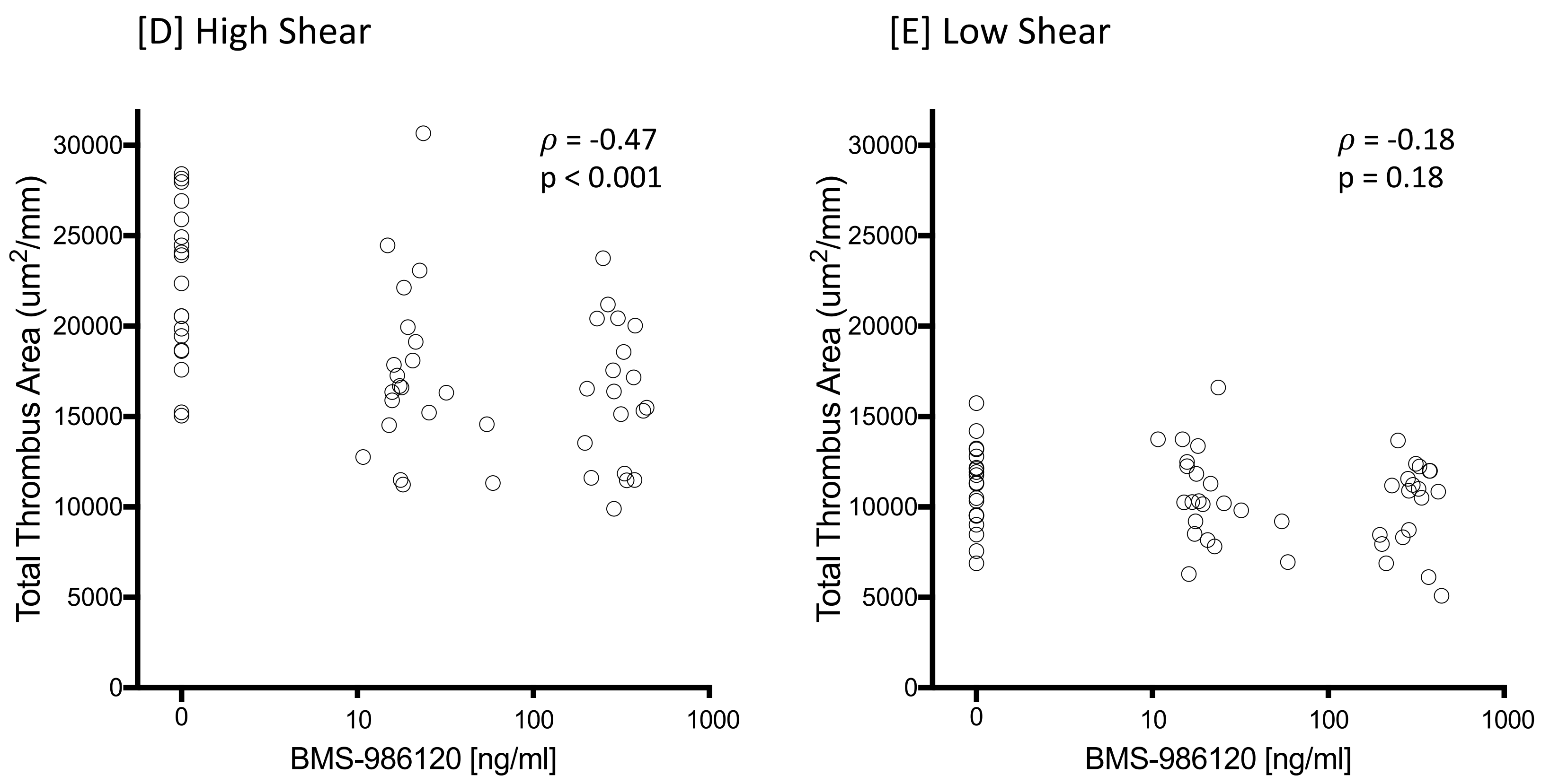

Figure II. Correlations between plasma concentrations of BMS-986120 and selected study endpoints.

Data shown are scatter plots of [A] PAR4 AP stimulated p-selectin expression, [B] PAR4 AP stimulated platelet-monocyte aggregates, [C] PAR4 AP stimulated platelet aggregation, [D] total thrombus area at high shear, and [E] total thrombus at low shear in volunteers randomised to BMS-986120. Correlation coefficients (ρ) and p-values were determined by Spearman's rank-order correlation.

Platelet Aggregation

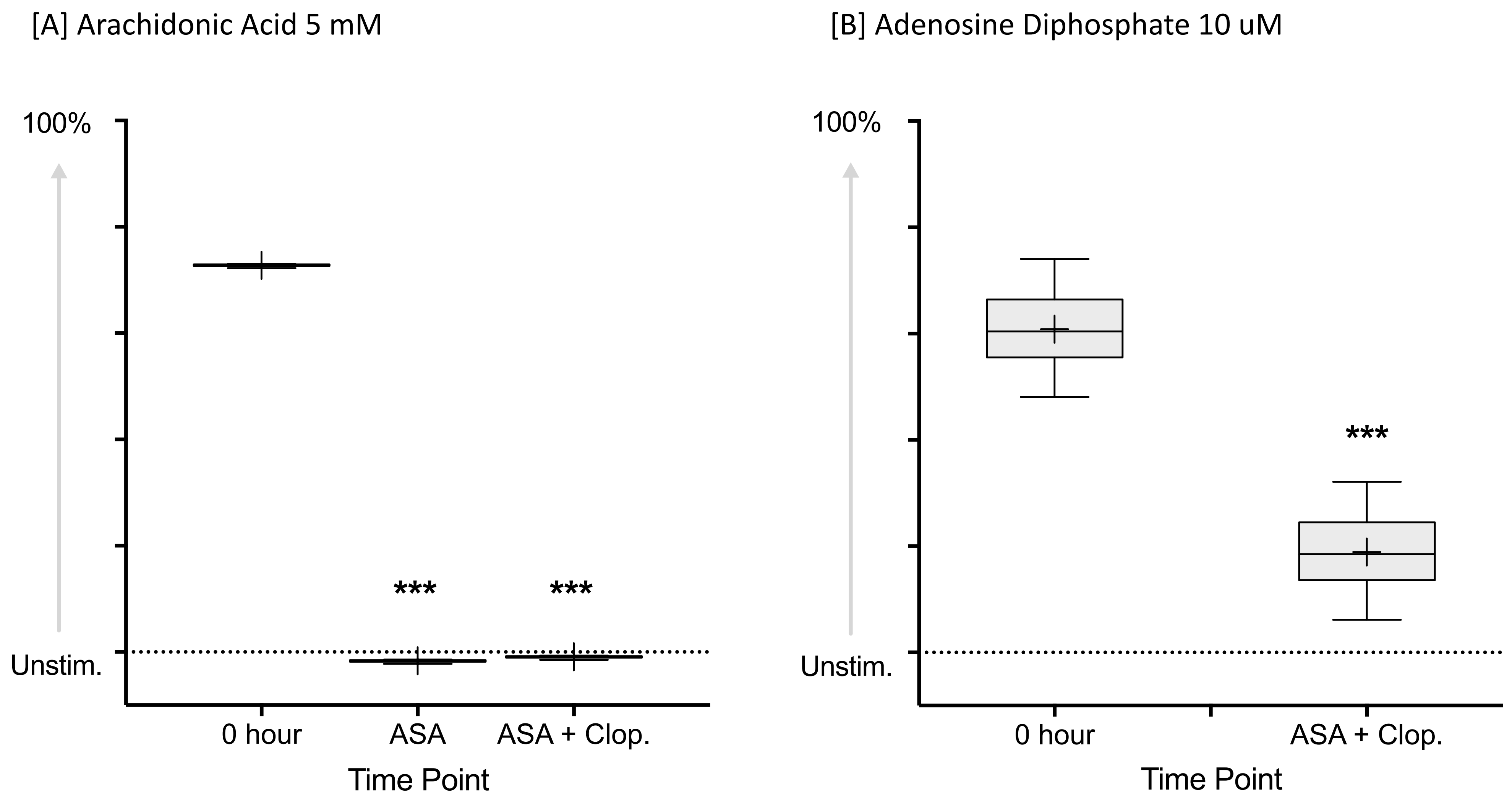

Figure III. Box plots of platelet aggregation in response to (A) arachidonic acid [5 mM] and (B) adenosine diphosphate [10 μM] in volunteers randomised to aspirin ± clopidogrel.

Data shown are the adjusted means (+) normalised to unstimulated values. The line within the box represents the median, upper and lower edges of the box represent the 75th and 25th percentiles, and upper and lower whiskers represent the 95th and 5th percentiles. Statistical comparisons (Least Significance Difference test) versus 0 hour are shown above each plot: * $p<0.05$, ** $p<0.01$, *** $p<0.001$.
