# Supplementary material for: PAR4 (Protease-Activated Receptor 4) Antagonism With BMS-986120 Inhibits Human Ex Vivo Thrombus Formation
Source: Arterioscler Thromb Vasc Biol. 2018 Jan 24;38(2):448–56. doi: 10.1161/ATVBAHA.117.310104 (PMC5779320; doi:10.1161/ATVBAHA.117.310104)
Supplement: Supplementary file 2 [file atv-38-448-s002.pdf]

SUPPLEMENTAL MATERIAL

Methods

Study Design

This was a phase I parallel group (n=20 per treatment arm) prospective randomized open-label blinded endpoint (PROBE) trial conducted at a single site (Clinical Research Facility, Royal Infirmary of Edinburgh, Scotland) between the 23rd September 2015 and 1st March 2016. *Ex vivo* platelet aggregation, platelet activation and thrombus formation were measured at 0 (pre-treatment), 2 and 24 h after oral administration of (a) 60 mg of BMS-986120 or (b) 600 mg aspirin with a second 600 mg aspirin and 600 mg clopidogrel at 18 h (Figure I in the online-only Data Supplement). Aspirin ± clopidogrel were included as a positive control and assay validation tool.

The trial was sponsored by Bristol-Myers Squibb (BMS) and was designed collaboratively with the host academic center. The study was approved by the local research ethics committee, conducted in accordance with the Declaration of Helsinki and with the written informed consent of all volunteers. Clinical Trial Authorization was provided by the Medicines and Healthcare products Regulatory Authority (MHRA) of the United Kingdom.

Study End-Points

The primary outcome was the effect of BMS-986120 on total thrombus area as compared to pre-treatment. Secondary and exploratory end-points included the effect of study drug (BMS-986120 or aspirin ± clopidogrel) on platelet aggregation, p-selectin expression, platelet-monocyte aggregates, and thrombus composition (platelet- and fibrin-rich thrombus area).

Study Population

Healthy non-smoking male and female volunteers between the ages of 18 and 65 years (inclusive) and with a body mass index (BMI) of 18 to 32 kg/m^2 underwent screening including detailed medical history, physical examination, laboratory blood tests, urinalysis and 12-lead electrocardiogram (ECG). Exclusion criteria were women of child-bearing potential and any clinically significant coexisting condition including hypertension, hyperlipidemia, diabetes mellitus, gastrointestinal disease that could affect drug absorption, coagulopathy, recent infective or inflammatory condition, known liver disease or screening blood tests indicative of renal, liver, clotting, thyroid or hematological abnormality. Volunteers must not have been taking any prescription medications for 4 weeks, over-the-counter medications, herbal supplements and vitamins for 1 week, and alcohol or caffeine containing products for 72 hours prior to and for the duration of the study.

Dose Selection

BMS-986120 is a competitive, reversible inhibitor of PAR4 AP induced platelet aggregation (K_{on}=0.12 ± 0.043 nM^{-1}min^{-1}, K_{off}=0.0082 ± 0.0016 min^{-1}, K_d=0.098 ± 0.016 nM). In cynomolgus monkeys, BMS-986120 demonstrated dose-dependent (0.2-1.0 mg/kg) preservation of carotid arterial flow following

electrolytic injury at the expense of a slight increase in mesenteric and kidney bleeding times[1]. In a single ascending (0.5-180 mg) and multiple ascending dose study (2.5-100 mg daily for up to 14 days) in healthy volunteers, BMS-986120 was found to be safe and well tolerated with complete and reversible inhibition of PAR4 agonist peptide (AP) stimulated platelet aggregation at ≥10 mg daily[2]. On the basis of these studies, a 60 mg dose was selected for the present phase 1 trial as this was calculated to be sufficient to inhibit platelet aggregation 2 h post dose and would be at the edge of a potential pharmacodynamic effect at 24 h. This would allow for "dose ranging" with a single dose of BMS-986120 whilst remaining well within the safety experience.

Doses of aspirin (600 mg) and clopidogrel (600 mg) were selected to reflect the maximal antithrombotic efficacy that might reasonably be expected in clinical practice following initiation of these antiplatelet agents in an acute setting.

Study Outcome Measures

Blood Sampling and Agonists

All blood samples for pharmacodynamic and pharmacokinetic assessments were drawn uncuffed through a 17G cannula in the ante-cubital fossa. For each time point, the first 2.5 mL of blood was discarded. PAR1 and PAR4 APs (SFLLRN and A-Phe(4-F)-PGWLVKNG respectively) were provided by Bristol-Myers Squibb (Princeton, USA), adenosine diphosphate (ADP) by Sigma-Aldrich (Gillingham, UK) and arachidonic acid (AA) by Alpha Laboratories (Eastleigh, UK).

Pharmacokinetic Assessment

Plasma concentrations of BMS-986120 were determined at 0, 1, 2, 3, 4, 5, 6, 9 and 24 h post treatment using a validated liquid chromatography tandem-mass spectrometry (LC-MS/MS) method with a lower limit of quantification (LLQ) of 0.250 ng/mL, with an accuracy coefficient of variation of <5 % and precision (intra- and inter-assay) coefficients of variation of <10 %. Blood samples were collected into 3 mL K_2EDTA vacutainers (Becton-Dickinson, Cowley, UK) and placed on wet ice. Within 1 h of collection, samples were centrifuged at 1200 *g* (2-8 °C) for 10 min. Plasma was decanted and stored at -20 °C before analysis.

Platelet Aggregation

Platelet aggregation was assessed by optical aggregometry (PAP-8E; Bio/Data Corp, Horsham, PA, USA) of platelet-rich plasma (PRP). To obtain PRP, 18 mL of blood was collected, mixed immediately with 2 mL of 3.8 % sodium citrate, and then centrifuged at 300 *g* (room temperature) for 15 min. For reference, 2 mL of PRP was centrifuged at 5500 *g* for 6 min to obtain platelet-poor plasma (PPP). All samples were allowed to equilibrate for 10 min (37 °C) after the addition of agonist and the peak aggregation recorded.

Platelet Activation

Platelet p-selectin expression and platelet-monocyte aggregates were determined by flow cytometry. Blood (5 mL) was collected into 50 μL of 75

mM D-phenylalanyl-L-propyl-L-arginine chloromethylketone (PPACK; Enzo Life Sciences, Exeter, UK) then immediately aliquoted into eppendorfs pre-filled with or without agonist and the following conjugated monoclonal antibodies: allophycocyanin (APC)-conjugated CD14, phycoerythrin (PE)-conjugated CD62P and fluorescein isothiocyanate (FITC)-conjugated CD42a (Becton-Dickinson). All antibodies were diluted 1:10. Samples were incubated for 20 min at room temperature before fixing with 1 % paraformaldehyde (p-selectin) or FACS-Lyse (Becton-Dickinson; platelet-monocyte aggregates). All samples were analysed within 24 h using a FACSCalibur flow cytometer (Becton-Dickinson). Data analysis was performed using FlowJo v10 (Treestar, Oregon, USA).

Ex Vivo Perfusion Model of Thrombosis

The effect of study compound on ex vivo thrombus formation was assessed using the Badimon perfusion chamber as previously described[3]. In brief, a pump was used to draw native (unanticoagulated) blood directly from an antecubital vein through a series of three cylindrical perfusion chambers maintained at 37°C in a water bath. Each chamber contained a strip of porcine aorta from which the intima and a thin layer of media had been removed. The ultrastructure of porcine aorta closely resembles that of human arteries and by removing the intima and a thin layer of media, blood is exposed to collagen fibres (type I and type III), proteoglycans, basement membrane, elastin, smooth muscle cells and other constituents common to an atherosclerotic plaque[4-8]. Rheological conditions in the first chamber were set to simulate those of patent medium-sized coronary arteries (inner lumen diameter, 2.0 mm; vessel wall shear rate, 212 s^{-1}; mean blood velocity, 5.3 cm/s; Reynolds number: 30), whereas those in the second and third chambers simulate those of mild to moderately stenosed coronary arteries (inner lumen diameter, 1.0 mm; vessel wall shear rate: 1690 s^{-1}; mean blood velocity, 21.2 cm/s; Reynolds number: 60). Shear conditions at the vessel wall were calculated from the expression for shear rate given for a Newtonian fluid in tube flow[9,10]. Each study lasted for exactly 5 min during which flow was maintained at a constant rate of 10 mL/min. All studies were performed using the same perfusion chamber and by the same operator.

Histomorphometric Analysis

As thrombus forms along the entire length of the exposed porcine aortic strip, the mean transverse cross-sectional area gives a reliable reflection of total thrombus[6]. Following fixation, the proximal and distal 1 mm of the exposed substrate were discarded and the remainder cut into eight segments. Individual segments were then embedded in paraffin wax from which 4-μm sections were prepared for histomorphometric analysis.

To detect total thrombus area, endogenous hydrogen peroxide activity was blocked using 3 % hydrogen peroxide solution (Leica Microsystems GmbH, Wetzlar, Germany) for 5 minutes. Sections were then incubated at room temperature for 1 hour with polyclonal rabbit anti-human fibrin(ogen) antibody (1.2 μg/mL, Dako, Glostrup, Denmark; Cat. No. A0080) and monoclonal mouse anti-human CD61 antibody (1.28 μg/ml, Dako; Cat. No. M0753). Antigen visualisation was performed using a Bond Polymer refine detection kit

(Leica Microsystems GmbH) and treatment with 3,3'-diaminobenzidine substrate chromogen (66 mM, Dako). Finally, sections were counterstained with a modified Masson's trichrome (hematoxylin and sirius red 0.1 %; Figure II in the online-only Data Supplement).

To examine the effect of study drug(s) on fibrin-rich and platelet-rich thrombus formation, endogenous hydrogen peroxide activity was blocked using 3 % hydrogen peroxide solution (VWR, Radnor, PA, USA) for 10 min and non-specific binding blocked using 20 % normal goat serum (Biosera, Nuaille, France) in Tris-Buffered Saline with 0.01% Tween (TBST)). Sections were then incubated with polyclonal rabbit anti-human fibrin(ogen) antibody (1.2 μg/ml) to detect fibrin and CD61 monoclonal mouse anti-human antibody (0.32 μg/ml) to detect platelets. Following TBST washes, goat anti-rabbit peroxidase (1:500; Abcam, Cambridge, UK) was applied and the presence of antigen visualized with Tyramide Cy3 (1:50; Perkin Elmer, Boston, MA, USA; Cat. no. NEL744B001KT) and FITC (1:50; Perkin Elmer, Waltham, MA, USA; Cat. no. NEL741B001KT) before nuclear counterstaining with DAPI (5 μg/ml; Sigma-Aldrich; Cat. No. D9542).

Prior to the first experimental sample, non-specific binding of the primary antibodies was excluded using tissue negative controls (perfusion chamber porcine sections exposed to saline rather than blood). To ensure staining for platelets and fibrin(ogen) antigen was the result of detection of the antigen, secondary antibody controls (with the primary antibody absent) were run in parallel for each volunteer. No labelling was observed.

A semi-automated slide scanner (Axioscan Z1; Zeiss, Jena, Germany) and image analysis software (Definiens, Munich, Germany) were used by a blinded operator to quantify thrombus area and composition. Digital images of each section were acquired at ×20 magnification. High-resolution classifiers based on colour were established to detect total thrombus, platelet and fibrin area.

Safety and tolerability
The primary safety end-point was the incidence of serious adverse events (SAEs) or death during and for up to 30 days post dosing. Adverse events (AEs) not meeting the SAE threshold were also recorded. All volunteers received telephone follow up on day 8. Reports of SAEs and AEs could originate from the volunteer, investigator or study personnel. Additional safety endpoints included changes in hematological and biochemical indices, hematuria (including microhematuria), alteration in the 12-lead electrocardiogram (ECG), or abnormal findings on physical examination performed at baseline, 2 and 24 h post dosing.

Statistical Analysis
Following study completion, the database was locked and all statistical analyses carried out independent of the sponsor. The demographic and baseline characteristics of volunteers are expressed as mean ± standard deviation (SD) for continuous variables and percentages for categorical variables. The effect of study drug(s) on endpoints was assessed by general

linear mixed effects models, with perfusion procedure (baseline, 2 and 24 h) as fixed effects and subjects as random effects. Mean within-subject differences for the change from baseline were generated and analysed using the Least Significance Difference (LSD) test. Prior to model fitting, total thrombus area, platelet area and fibrin area were log-transformed. Associations between plasma concentrations of BMS-986120 and study end-points were determined by Spearman's rank-order correlation (*ρ*). Two sided p-values of ≤0.05 were considered statistically significant. Analyses were performed using SPSS version 21.0 (IBM Corp., Armonk, New York) and R version 3.3.1 (R Project for Statistical Computing, Vienna, Austria).

References

1. Wong PC, Seiffert D, Bird JE et al. Blockade of protease-activated receptor-4 (PAR4) provides robust antithrombotic activity with low bleeding. *Sci Transl Med*. 2017;9(371):eaaf5294.

2. Ismat FA, Ma X, Wang Z, Frost CE, Ni YG, Yang J. Abstract TMP91: Phase I Assessment of the Safety, Tolerability, Pharmacokinetics and Pharmacodynamics of the Oral Protease-activated Receptor-4 Antagonist BMS-986120. *Stroke*. 2016;47(Suppl 1):ATMP91.

3. Lucking AJ, Chelliah R, Trotman AD, Connolly TM, Feuerstein GZ, Fox KA, Boon NA, Badimon JJ, Newby DE. Characterisation and reproducibility of a human ex vivo model of thrombosis. *Thrombosis Research*. 2010;126(5):431-435.

4. Badimon L, Badimon JJ, Turitto VT, Vallabhajosula S, Fuster V. Platelet thrombus formation on collagen type I. A model of deep vessel injury. Influence of blood rheology, von Willebrand factor, and blood coagulation. *Circulation*. 1988;78(6):1431-1442.

5. Badimon L, Badimon JJ. Mechanisms of arterial thrombosis in nonparallel streamlines: platelet thrombi grow on the apex of stenotic severely injured vessel wall. Experimental study in the pig model. *J Clin Invest*. 1989;84(4):1134-1144.

6. Dangas G, Badimon JJ, Coller BS, Fallon JT, Sharma SK, Hayes RM, Meraj P, Ambrose JA, Marmur JD. Administration of Abciximab During Percutaneous Coronary Intervention Reduces Both Ex Vivo Platelet Thrombus Formation and Fibrin Deposition : Implications for a Potential Anticoagulant Effect of Abciximab. *Arterioscler Thromb Vasc Biol*. 1998;18(8):1342-1349.

7. Chow M-J, Turcotte RL, Lin CP, Zhang Y. Arterial Extracellular Matrix: A Mechanobiological Study of the Contributions and Interactions of Elastin and Collagen. *Biophysical Journal*. 2014;106(12):2684-2692.

8. Tonar ZK, Kub kov T, Prior C, Demj n E, Li ka VC, Kr l kov M, Witter K. Segmental and age differences in the elastin network, collagen, and smooth muscle phenotype in the tunica media of the porcine aorta. *Annals of Anatomy - Anatomischer Anzeiger*. 2015;201:79-90.

9. Schechter RS. Transport Phenomena (Bird, R. Byron; Stewart, Warren E.; Lightfoot, Edwin N.). *J Chem Educ*. 1961;38(9):A640.

10. Badimon L, Padro T, Vilahur G. Extracorporeal Assays of Thrombosis. In: *Platelets and Megakaryocytes*. Vol 788. Methods in Molecular Biology. New York, NY: Springer New York; 2011:43-57.
