# Supplementary figures and images for: PAR4 (Protease-Activated Receptor 4) Antagonism With BMS-986120 Inhibits Human Ex Vivo Thrombus Formation
Source: Arterioscler Thromb Vasc Biol. 2018 Jan 24;38(2):448–56. doi: 10.1161/ATVBAHA.117.310104 (PMC5779320; doi:10.1161/ATVBAHA.117.310104)

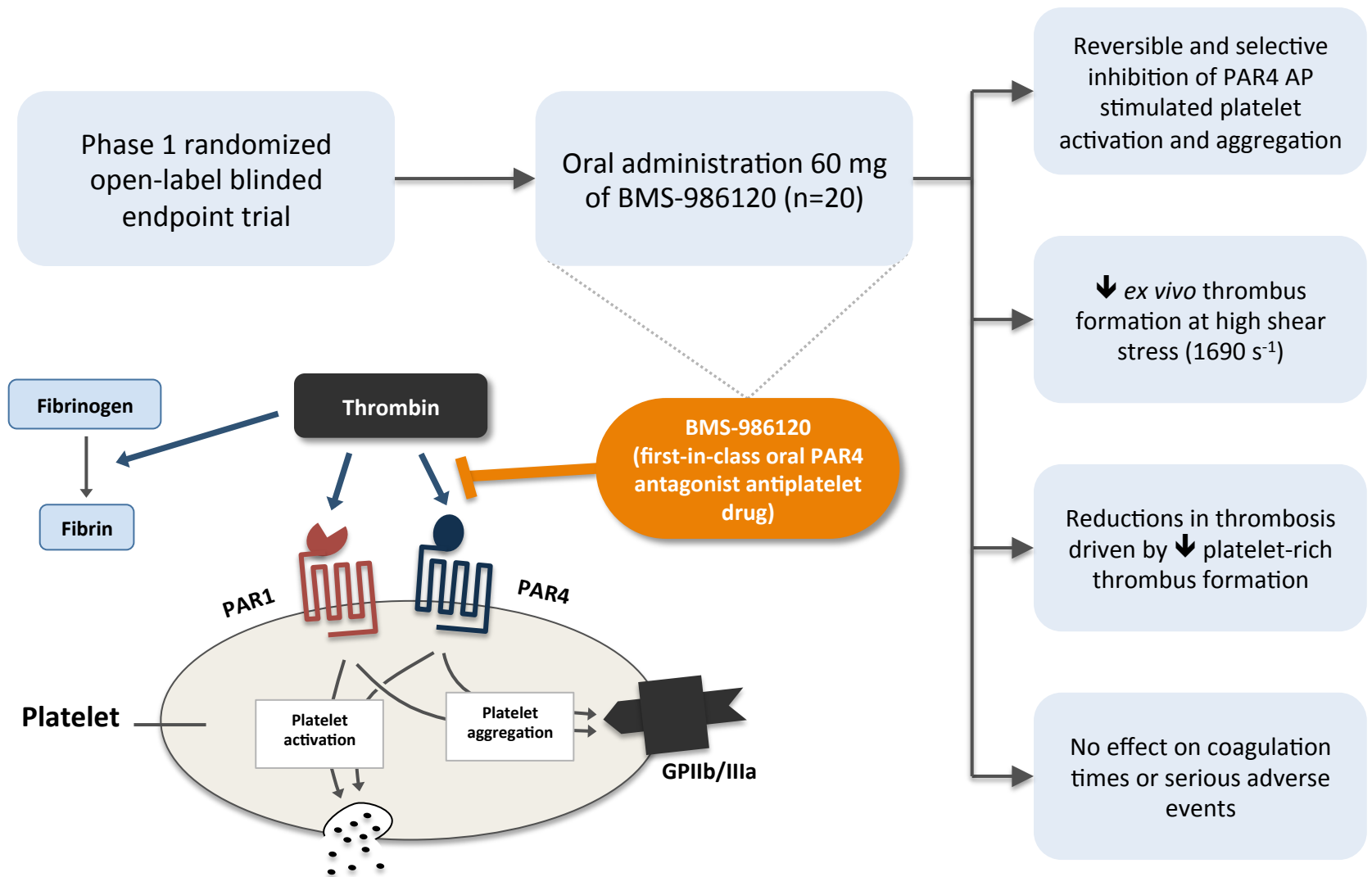

Supplement: Supplementary file 3 [file atv-38-448-s003.pdf]
